# Ceftriaxone-Induced Hemolytic Anemia: A Rare and Fatal Reaction

**DOI:** 10.7759/cureus.59646

**Published:** 2024-05-04

**Authors:** Michael V Dicaro, Claire Chen, Shawn Wang, Annette Y Eom, Sandhya Wahi-Gururaj

**Affiliations:** 1 Internal Medicine, Kirk Kerkorian School of Medicine at University of Nevada Las Vegas (UNLV), Las Vegas, USA

**Keywords:** hemolytic anemia, medical icu, critical care, infectious disease medicine, pharmacology, id critical care

## Abstract

Ceftriaxone, a regularly used antibiotic for broad-spectrum coverage, is a rare cause of hemolytic anemia. Patients may present with truncal pain, nausea, vomiting, and an acute drop in hemoglobin within 48 hours of administration. Prompt recognition and initiation of treatment are essential. We describe a case of a 65-year-old woman being treated for osteomyelitis who developed hemolytic anemia, disseminated intravascular coagulation, and multi-system organ failure after being de-escalated from cefepime to ceftriaxone.

## Introduction

Ceftriaxone (CTX) is a popular third-generation cephalosporin due to its broad gram-positive and gram-negative bacterial coverage profile and convenient dosing. While the medication is generally well-tolerated, it can infrequently produce severe reactions, including anaphylaxis, angioedema, and hemolytic anemia [1,2]. Ceftriaxone-induced hemolytic anemia (CIHA) is a rare phenomenon, with an incidence of roughly 1/1,000,000 [1,3]. Recognition and management are essential, as CIHA carries a mortality rate of 30% to 50%. Clinically, patients present with nausea, vomiting, and truncal pain with a rapid decline in hemoglobin and multisystem organ failure within 48 hours of CTX administration. Here, we describe a rare and fatal case of CIHA in a 65-year-old female patient who was de-escalated from cefepime to CTX. This case was previously presented as a poster presentation at the American College of Chest Physicians annual meeting on October 9th, 2023.

## Case presentation

A 65-year-old woman with a past medical history of type 2 diabetes mellitus and left foot osteomyelitis requiring recent amputation of the left 4th and 5th metatarsals, as well as six weeks of antibiotic therapy with doxycycline and amoxicillin/clavulanate, presented to the hospital with a two-day history of left medial foot pain and a low-grade fever of 100.2°F. An MRI revealed osteomyelitis of the left first metatarsal bone. The patient initially improved with vancomycin and cefepime. A surgical consultation was obtained for further evaluation; the patient was not amenable to further amputation or debridement. Infectious disease specialists were also consulted, and the patient was recommended daptomycin 8 mg/kg and CTX 2g daily for six weeks. 

Five minutes after receiving her first dose of CTX, the patient developed nausea, vomiting, and crushing substernal pain radiating to the back. Ceftriaxone was discontinued immediately. A STAT chest X-ray (CXR), electrocardiogram (EKG), and CT angiography (CTA) of the abdomen and pelvis were unremarkable. The STAT labs revealed an elevated troponin level of 564 ng/mL and a significant drop in hemoglobin from 10.3 g/dL to 6.8 g/dL. A drug-induced hemolytic anemia was suspected; therefore, direct anti-globulin tests for C3 and IgG were ordered. The patient was given 100 mg of intravenous methylprednisolone and two units of packed red blood cells.

**Figure 1:**
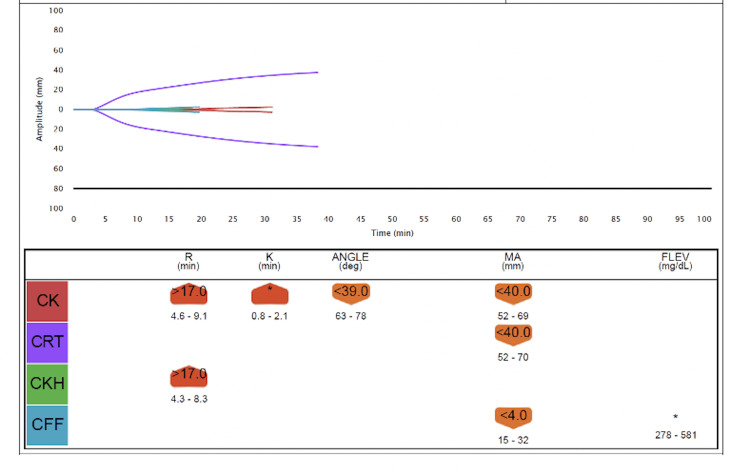
Thromboelastography analysis no.1 at 12:17 a.m. on ICU day 1 CK: Citrated kaolin, CRT: Citrated rapid TEG, CKH: Citrated kaolin with heparinase, CFF: Citrated functional fibrinogen, R: Reaction time in minutes, K: The speed of the formation of the clot from R time to a specific clot strength, Angle: The speed of the clot strengthening, MA: Maximum amplitude, FLEV: Functional fibrinogen, TEG: Thromboelastography

**Figure 2:**
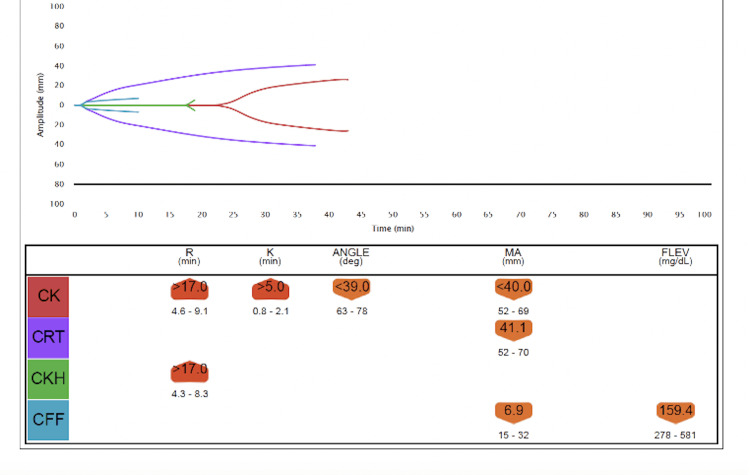
Thromboelastography analysis no.2 at 17:56 p.m. on ICU day 1 CK: Citrated kaolin, CRT: Citrated rapid TEG, CKH: Citrated kaolin with heparinase, CFF: Citrated functional fibrinogen, R: Reaction time in minutes, K: The speed of the formation of the clot from R time to a specific clot strength, Angle: The speed of the clot strengthening, MA: Maximum amplitude, FLEV: Functional fibrinogen, TEG: Thromboelastography

**Figure 3:**
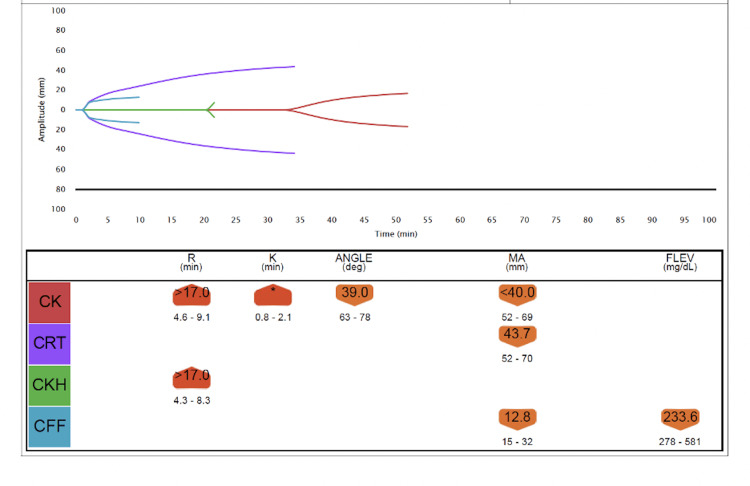
Thromboelastography analysis no.3 at 00:03 a.m. on ICU day 2 CK: Citrated kaolin, CRT: Citrated rapid TEG, CKH: Citrated kaolin with heparinase, CFF: Citrated functional fibrinogen, R: Reaction time in minutes, K: The speed of the formation of the clot from R time to a specific clot strength, Angle: The speed of the clot strengthening, MA: Maximum amplitude, FLEV: Functional fibrinogen, TEG: Thromboelastography

**Figure 4:**
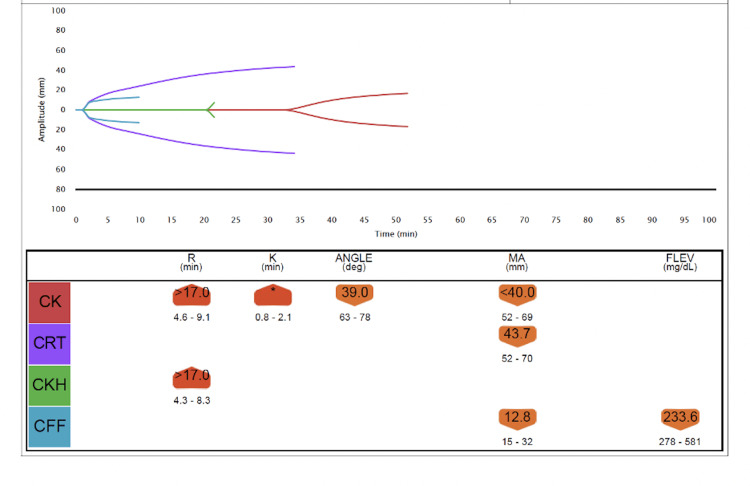
Thromboelastography analysis no. 4 at 07:42 a.m. on ICU day 2 CK: Citrated kaolin, CRT: Citrated rapid TEG, CKH: Citrated kaolin with heparinase, CFF: Citrated functional fibrinogen, R: Reaction time in minutes, K: The speed of the formation of the clot from R time to a specific clot strength, Angle: The speed of the clot strengthening, MA: Maximum amplitude, FLEV: Functional fibrinogen, TEG: Thromboelastography

**Figure 5:**
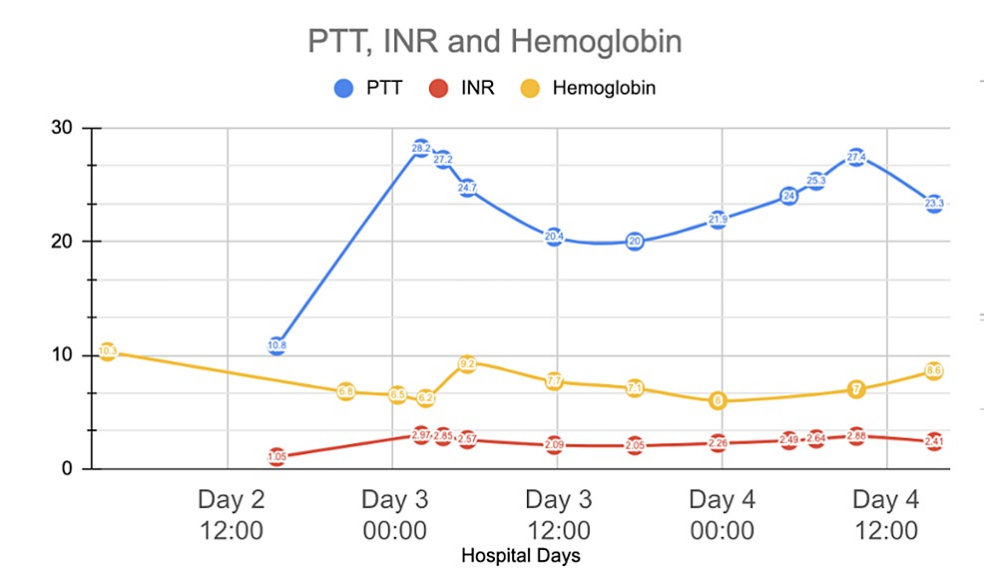
The PTT, INR, and hemoglobin trends during hospitalization PTT: Partial thromboplastin time, INR: International normalized ratio

Over the next several hours, the patient deteriorated rapidly. She developed hypotension, confusion, and acute hypoxic respiratory failure. Her oxygen requirements increased until she required a 70% fraction of inspired oxygen (FiO2) on non-invasive positive-pressure ventilation (NIPPV). On hospital day 3, she was upgraded to the ICU for increased oxygen requirements and vasopressor support. Labs were noted to repeatedly hemolyze, delaying a timely assessment of the extent of hemolysis. However, subsequent labs revealed hemoglobin persistently below 7 g/dL despite transfusion. The international normalized ratio (INR) was elevated at 2.5, and fibrinogen was < 50 mg/dL. Thromboelastography (TEG) revealed an elevated K time greater than 5 and a decreased angle (Figure 1). The patient was treated for disseminated intravascular coagulation (DIC) with fresh frozen plasma, and repeat TEGs were ordered to guide further management with blood products (Figures 2-4). Prothrombin time, INR, and hemoglobin trends throughout the patient’s hospitalization are shown in Figure 5.

The patient’s hypoxic respiratory failure continued to worsen. On hospital day 3 and ICU day 1, she required intubation due to the increased work of breathing on the nasal intermittent positive pressure ventilation (NIPPV). She developed oliguric renal failure and shock liver, with a creatinine of 3.74, blood urea nitrogen (BUN) of 56, potassium of 7.2, estimated glomerular filtration rate (eGFR) of 12, and aspartate aminotransferase (AST)/alanine transaminase (ALT) of 5335/1213. Her PT and INR were 27.4 and 2.88, respectively. Given her oliguric renal failure and acidosis with a pH of 7.01, bicarbonate infusion and continuous renal replacement therapy were initiated. A diagnosis of autoimmune hemolytic anemia due to CTX was made. She was started on aggressive therapy with IV methylprednisolone and IV immunoglobulin with a plan to begin plasmapheresis, as per the standard of care for severe autoimmune hemolytic anemia. Despite these measures, she continued to deteriorate. The patient’s multi-system organ failure continued to worsen until a family discussion was held. The patient was transitioned to comfort measures before succumbing to her illness. She was pronounced deceased three days after CTX administration. A direct antiglobulin test (DAT) for C3 and IgG posthumously tested positive, confirming the diagnosis of CIHA.

## Discussion

More than 130 different drugs are implicated in immune hemolytic anemia [4]. Common inducers of drug-induced immune hemolytic anemia (DIHA) include antimicrobials, which represent roughly 42% of all DIHA cases; non-steroidal anti-inflammatories (16%); antineoplastics (13%); and antihypertensives or diuretics (6%) [4]. Most commonly, cephalosporins and penicillins are implicated. Specifically, CIHA has an incidence of one in a million, with mortality rates as high as 50% due to its rapid progression [1]. Ceftriaxone-induced hemolytic anemia has been noted to have more severe symptoms and a higher mortality rate than other forms of DIHA [5]. Clinically, patients present with nausea, vomiting, and truncal pain within 48 hours of CTX administration. They will display an acute drop in hemoglobin and commonly develop acute renal failure as the kidney experiences both prerenal ischemia and nephrotoxic pigment deposition [1,6] as occurred in our patient. In most reported cases of CIHA, CTX binds to RBC membranes, forming haptens that provoke complement activation, antibody synthesis, and drug-dependent immune complex deposition that induce intravascular hemolysis [1,3,7-9]. In one study involving 25 patients with CIHA, C3 complement was detected on the surface of the RBC membranes, and C3 complement as well as antibodies were detected in the serum in 100% of cases [10]. Notably, IgM antibodies were usually detected, but IgG antibodies were also frequently seen.

Our patient had an acute reaction to CTX after receiving several doses of cefepime without complications. Cross-reactivity between cephalosporins has not been adequately studied; however, several studies show a >40% cross-reactivity rate [5,11]. These reactions occur against the R1 side chain and, less commonly, the R2 chain. One case in 2020 reported a similar example of cephalosporin cross-reactivity where a patient was potentially sensitized by cefepime 10 months prior to a fatal episode of CTX-induced anaphylactic shock [12]. Similarly, our patient may have been sensitized to CTX after cefepime administration, thereby developing antibodies to the R1 side chain. 

Thromboelastography is a type of non-invasive test that quantitatively measures the body’s ability to form a clot, providing real-time metrics of a patient’s in vitro hemostatic state. The utilization of TEG allowed us to understand the dynamic changes in the patient’s viscoelastic properties as the severity of hemolytic anemia escalated. The TEG results were collected hours after the administration of CTX and showed early signs of DIC, while the final reading demonstrates the progression to late DIC. The co-occurrence of DIHA and DIC has been detailed before, with a 2013 study that hypothesizes a possible link for DIHA occurring before DIC as (1) platelets contain lipids necessary for activation of the coagulation cascade, and (2) immune complexes triggering coagulation can be taken up by the reticuloendothelial system (RES) thereby decreasing the capacity of RES to clear procoagulant present in the blood.

The current standard-of-care management of CIHA involves the immediate cessation of CTX. Resuscitation with RBC transfusions should be initiated as needed. Patients with CIHA may be prone to consumptive coagulopathy, and if suspected, appropriate resuscitation with platelets and coagulation factor concentrates should be initiated. Steroids may be used in refractory cases in patients with severe hemolysis [13,14], as well as other immunosuppressants, including cyclophosphamide and azathioprine [1,13]. Plasmapheresis may also be considered in refractory cases, specifically in patients with renal failure [1]. 

## Conclusions

Ceftriaxone, a commonly used antibiotic, can rarely result in drug-induced hemolytic anemia. It can occur as a standalone reaction to CTX or during de-escalation from other cephalosporin medications. Ceftriaxone-induced hemolytic anemia has the potential to trigger DIC, shock, multisystem organ failure, and death. If CIHA is suspected, prompt diagnosis and intervention should be pursued. Ceftriaxone should be stopped immediately, resuscitation with appropriate blood products should be initiated, and immunosuppressants may be used in severe cases.
